# Supplementary material for: Deficiency in Dipeptidyl Peptidase-4 Promotes Chemoresistance Through the CXCL12/CXCR4/mTOR/TGFβ Signaling Pathway in Breast Cancer Cells
Source: Int J Mol Sci. 2020 Jan 26;21(3):805. doi: 10.3390/ijms21030805 (PMC7037814; doi:10.3390/ijms21030805)
Supplement: Supplementary file 1 [file ijms-21-00805-s001.pdf]

Supplementary Materials:

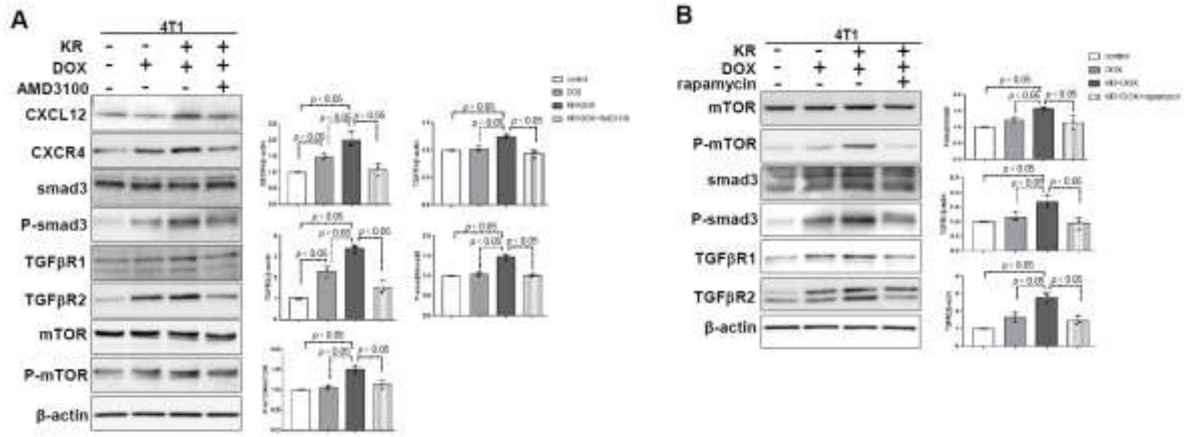

Figure S1. (**A**) Western blot analysis of CXCL12, CXCR4, phosphorylation smad3 (P-smad3), TGFR1, TGFR2 and phosphorylation mTOR (P-mTOR) in 4T1 cells pretreated with KR62436 (50 μmol/L) for 48 h and then treated with or without DOX (0.425 μmol/L) in the presence or absence of AMD3100 (30 μmol/L) for another 48 h. (**B**) Western blot analysis of phosphorylation smad3 (P-smad3), TGFR1 and TGFR2 in 4T1 cells in 4T1 cells pretreated with KR62436 (50 μmol/L) for 48 h and then treated with or without DOX (0.425 μmol/L) in the presence or absence of rapamycin (1 μmol/L) for another 48 h. All densitometric analysis of CXCL12, CXCR4, TGFR1 and TGFR2 protein expression relative to b-actin levels, P-smad3 protein expression relative to smad3 levels and P-mTOR protein expression relative to mTOR (n = 3, per group) were performed by using ImageJ.

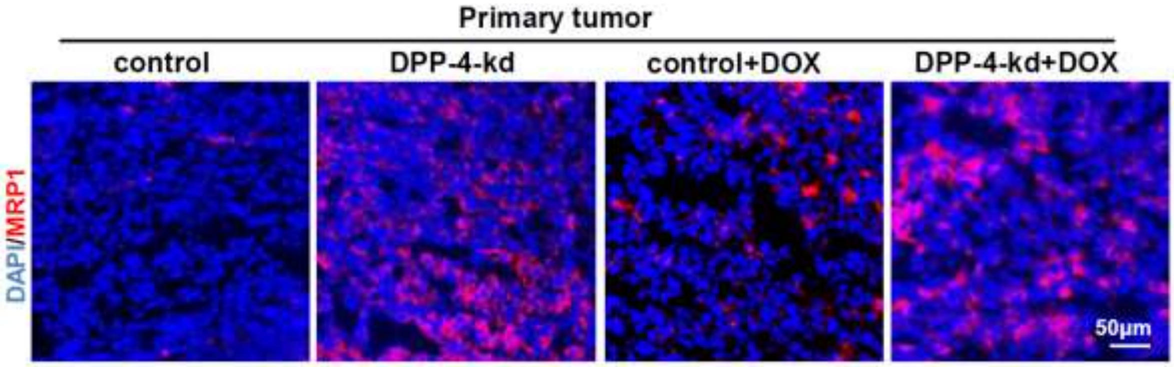

Figure S2. Immunofluorescence analysis of MRP1 expression in the primary tumors. For each group, representative images of six different fields of view at 200× magnification were evaluated. The scale bar indicated 50 μm each panel.
